# Associations Between Chronic Health Conditions and COVID-19 Preventive Behaviors Among a Nationally Representative Sample of U.S. Adults: An Analysis of the COVID Impact Survey

**DOI:** 10.1089/heq.2020.0031

**Published:** 2020-08-05

**Authors:** Marlene Camacho-Rivera, Jessica Y. Islam, Denise C. Vidot

**Affiliations:** ^1^ Department of Community Health Sciences, School of Public Health, SUNY Downstate Health Sciences University, Brooklyn, New York, USA.; ^2^ University of North Carolina Lineberger Cancer Center, Chapel Hill, North Carolina, USA.; ^3^ University of Miami School of Nursing and Health Studies, Coral Gables, Florida, USA.

**Keywords:** COVID-19, health disparities, chronic disease

## Abstract

**Purpose:** In the United States, over 2 million cases of COVID-19 cases have been identified and more than 100,000 lives have been lost. While COVID-19 related disparities among those with chronic conditions have been observed, research regarding the uptake of COVID-related preventive behaviors is scarce.

**Methods:** We utilized data from a sample of 2190 U.S. adults from the COVID-19 Impact Survey to examine associations between the presence of underlying chronic health conditions and COVID-19-related preventive behaviors (e.g., use of face masks, hand washing, social distancing, etc.). We used multivariable logistic regression models to model associations between COVID-19 preventive behaviors across demographic and health characteristics.

**Results:** Adults with cardiometabolic disease were more likely to report staying home because they felt unwell, compared with individuals without cardiometabolic disease. Individuals with underlying respiratory conditions were more likely to work from home, compared with individuals without a respiratory condition. Adults with immune conditions were twice more likely to report wearing a face mask when compared with individuals without immune conditions.

**Conclusion:** This study provides U.S. national prevalence estimates and differences in adherence to COVID-19 preventive behaviors among those with and without the presence of underlying chronic health conditions. The prevalence of key preventive measures was high in the overall sample. Yet, engagement in COVID-19-related preventive behaviors varied significantly across chronic disease conditions. Messages around continued maintenance of the behaviors should be reinforced. Study implications suggest a need for more targeted messaging and resources available for individuals with certain underlying chronic conditions.

## Introduction

On February 11, 2020, the World Health Organization announced a name for the new coronavirus: COVID-19, caused by the severe acute respiratory syndrome coronavirus 2 (SARS-CoV-2).^1^ Since the declaration, in the United States, over 2 million cases of COVID-19 cases have been identified and more than 100,000 lives have been lost.^2^ Public health strategies to reduce the transmission of COVID-19 have included enactment of policies such as quarantine and social distancing, closures of nonessential businesses, and recommendation of behaviors such as hand washing and wearing of face masks.^3,4^

Popular media and emerging studies have reported that implementation of policies and adherence to preventive best practices have varied widely geographically and by demographic subgroups.^5,6^ Simultaneously, public health and clinical researchers have reported the disproportionate burden of COVID-19 morbidity and mortality among racial and ethnic minorities, the elderly, and individuals with pre-existing chronic health conditions.^7–13^ While adherence to preventive behaviors may reduce the risk of COVID-19 morbidity, studies examining the uptake of recommended behaviors among the general U.S. population are scarce.

In this study, we aimed to identify differences in uptake to COVID-19 preventive behaviors, particularly as it relates to the presence of underlying chronic health conditions, as well as demographic characteristics, such as, gender, age, education, and race/ethnicity. To accomplish these study goals, we leverage publicly available data from the COVID-19 Impact Survey, a nationally representative sample of the U.S. adult population, designed to offer national insights about the American population's experiences, including health, economic, and social wellbeing questions, during the COVID-19 pandemic.

## Methods

### COVID-19 impact survey

Data for these analyses were obtained from the publicly available COVID-19 Household Impact Survey, conducted by the nonpartisan and objective research organization NORC at the University of Chicago for the Data Foundation. The COVID-19 Household Impact Survey is a philanthropic effort to provide national and regional COVID-19 statistics about health, social, and economic sequelae in the United States. The survey is designed to provide weekly estimates of the U.S. adult (ages 18 and older) household population nationwide and for 18 regional areas, including 10 states (CA, CO, FL, LA, MN, MO, MT, NY, OR, and TX) and 8 Metropolitan Statistical Areas (Atlanta, Baltimore, Birmingham, Chicago, Cleveland, Columbus, Phoenix, and Pittsburgh).

### AmeriSpeak sample

Funded and operated by NORC at the University of Chicago, AmeriSpeak^®^ is a probability-based panel designed to be representative of the U.S. household population. During the initial recruitment phase of the AmeriSpeak panel, randomly selected U.S. households were sampled using area probability and address-based sampling, with a known, nonzero probability of selection from the NORC National Sample Frame. These sampled households were then contacted by U.S. mail, telephone, and field interviewers. The panel provides sample coverage of ∼97% of the U.S. household population. Those excluded from the sample include people with P.O. Box only addresses, some addresses not listed in the USPS Delivery Sequence File, and some newly constructed dwellings. While most AmeriSpeak households participate in surveys by web, noninternet households were able to participate in AmeriSpeak surveys by telephone. Interviews were conducted in English and Spanish. Interviews were conducted with adults age 18 and overrepresenting the 50 states and the District of Columbia. Panel members were randomly drawn from AmeriSpeak. In households with more than one adult panel member, only one was selected at random for the sample. Invited panel members were given the option to complete the survey online or by telephone with an NORC telephone interviewer. The participation percent of invited panelists (*n*=11,133) was 19.7%. Out of the 2190 interviews completed, 2053 (94%) were completed online and 137 (6%) were completed by phone. Panelists were offered a small monetary incentive for completing the survey (amount not disclosed by NORC). The survey field period was April 20–26, 2020. The analytic sample includes 2190 adults nationwide. The final analytic sample were weighted to reflect the U.S. population of adults 18 years of age and over. The demographic weighting variables were obtained from the 2020 Current Population Survey. The count of COVID-19 deaths by county was obtained from USA Facts. As the analysis of publicly available data does not constitute human subjects research as defined at 45 CFR 46.102, the study did not require IRB review.

### Primary outcomes

The primary outcomes for this analysis was participants' responses (yes/no) to the following questions: “Which of the following measures, if any, are you taking in response to the coronavirus?” Of the 19 options, participants were able to select all that apply: Canceled a doctor appointment; Worn a face mask; Visited a doctor or hospital; Canceled or postponed work activities; Canceled or postponed school activities; Canceled or postponed dentist or other appointment; Avoided some or all restaurants; Worked from home; Studied from home; Canceled or postponed pleasure social or recreational activities; Avoided public or crowded places; Prayed; Avoided contact with high-risk people; Washed or sanitized hands; Kept six feet distance from those outside my household; Stayed home because I felt unwell; Wiped packages entering my home.

### Primary predictor

The primary predictor for this analysis was participants' self-report of a chronic health condition. Participants were then asked to reply “yes, no, or not sure” to the following question: “Has a doctor or other health care provider ever told you that you have any of the following: Diabetes; High blood pressure or hypertension; Heart disease, heart attack or stroke; Asthma; Chronic lung disease or chronic obstructive pulmonary disease (COPD); Bronchitis or emphysema; Allergies; a Mental health condition; Cystic fibrosis; Liver disease or end-stage liver disease; Cancer; a Compromised immune system; or Overweight or obesity.” Health conditions were aggregated into the following six categories: cardiometabolic (diabetes, high blood pressure, heart disease, heart attack or stroke, liver disease or end-stage liver disease), overweight/obesity, respiratory (asthma, chronic lung disease or COPD, bronchitis or emphysema), allergies, immune-related (cystic fibrosis, cancer, a compromised immune system), and mental health conditions.

### Covariates

The following covariates were included in the multivariable analyses: age categories (18–29, 30–44, 45–59, 60+), gender (male, female), race/ethnicity categories (non-Hispanic White, non-Hispanic Black, Hispanic, non-Hispanic Other), and education categories (no high school diploma, high school graduate or equivalent, some college, BA or above).

### Data analyses

Descriptive statistics are displayed in percentages among all respondents, unless otherwise labeled, and include a margin of error of +/− 3.0 percentage points at the 95% confidence intervals (CIs) among all adults. Chi-squared tests were used for univariate comparison of categorical variables, including gender, race/ethnicity, education level, age, and comorbidities. Logistic regression was used to calculate the odds ratios and 95% CIs associated with reporting “yes” to COVID-19 preventive behavior individually by the following six categories of chronic conditions: cardiometabolic, respiratory, overweight or obese, allergies, immune diseases, and mental health conditions. For the logistic regression analysis, those who were unexposed in our binary outcomes were those who answered no to the specific preventive behavior. The multivariable logistic regression models included adjustments for age, gender, race/ethnicity, and education. The type I error was maintained at 5%. Based on the exploratory nature of this analysis, we did not include an adjustment for multiple comparisons.^14,15^ All statistical analyses were conducted using Stata IC 15 (StataCorp LLC, College Station, TX). Sampling weights were applied to provide results that were nationally representative of the U.S. adult population.

## Results

Table 1 displays the descriptive characteristics of the analytic sample. Sixty-three percent of respondents were non-Hispanic White, 12% non-Hispanic Black, 17% Hispanic, and 9% were of another non-Hispanic race or ethnicity. Ten percent of respondents had less than a high school diploma. Self-report of adherence to COVID-19 preventive behaviors ranged from 8% among respondents who reported visiting a doctor or hospital to 92% who reported washing or sanitizing their hands. While the majority reported canceling social or recreational activities (69%), avoiding public or crowded places (80%), or avoiding some or all restaurants (72%), only 32% of participants reported working from home. The most common chronic health conditions reported were allergies (45%), cardiometabolic diseases (51%), and overweight or obesity (33%).

**Table 1.** Demographic Characteristics and COVID-19 Preventive Behaviors Among COVID Impact Survey Participants (*n*=2190)

	Prevalence, %		Prevalence, %
Age		COVID-19 preventive behaviors	
18–29	21.0	Worn a face mask	78.0
30–44	25.0	Visited a doctor or hospital	8.0
45–59	24.0	Canceled or postponed work activities	32.0
60+	30.0	Canceled or postponed school activities	21.0
Gender		Avoided some or all restaurants	72.0
Male	48.0	Worked from home	32.0
Female	52.0	Studied from home	15.0
Race/Ethnicity		Canceled or postponed social activities	69.0
White, NH	63.0	Avoided public or crowded places	80.0
Black, NH	12.0	Prayed	56.0
Hispanic	17.0	Avoided contact with high-risk people	62.0
Other, NH	9.0	Washed or sanitized hands	92.0
Education		Kept six feet distance from outsiders	85.0
No HS diploma	10.0	Stayed home because I felt unwell	11.0
HS graduate	28.0	Wiped packages entering my home	45.0
Some college	28.0		
Bachelors or above	34.0		
Chronic health conditions			
Diabetes	11.0		
High blood pressure/hypertension	32.0		
Heart disease, heart attack, stroke	8.0		
Asthma	13.0		
Chronic lung disease or COPD	5.0		
Bronchitis or emphysema	12.0		
Allergies	45.0		
A mental health condition	15.0		
Cystic fibrosis	1.0		
Liver disease	1.0		
A compromised immune system	7.0		
Overweight or obesity	33.0		

COPD, chronic obstructive pulmonary disease; HS, high school; NH, non-Hispanic.

### Prevalence of chronic health conditions by demographic characteristics

Table 2 displays differences in the prevalence of reported chronic health conditions by social and demographic characteristics. Significant differences in the prevalence of cardiometabolic diseases were observed across age categories (*p*<0.001), gender (*p*=0.023), and categories of race/ethnicity (*p*=0.005). Significant differences in the prevalence of overweight or obesity were observed between men and women (28% compared with 36%, respectively, *p*=0.01). Significant differences in the prevalence of respiratory diseases were observed across educational categories, with reported prevalence ranging from 19% among adults with a Bachelor's degree or more to 34% among adults with less than a high school degree (*p*=0.04), and between men and women (19% and 27%, respectively, *p*=0.003). Significant differences in the prevalence of allergies were reported between men and women, with a prevalence of 39% among men and a prevalence of 49% among women (*p*=0.003). Significant differences in the prevalence of immune-related diseases were observed across age categories, ranging from 5% among individuals in the 18–29 group to 20% among adults in the 60 or older age category (*p*<0.001). Significant differences in the prevalence of mental health conditions were observed across age categories (*p*<0.001) and between men and women (10% compared with 18%, respectively, *p*<0.001).

**Table 2.** Demographic Characteristics by Chronic Disease Condition

Total prevalence	Cardiometabolic diseases		Overweight/Obesity		Respiratory diseases		Allergies		Immune-related diseases		Mental health	
	37%	95% CI	33%	95% CI	24%	95% CI	45%	95% CI	13%	95% CI	15%	95% CI
	%		%		%		%		%		%	
Age												
18–29	13.5	8.1–21.6	27.4	20.4–35.7	25.1	18.3–33.4	41.2	32.9–50.0	5.7	2.9–10.9	23.8	17.3–31.8
30–44	18.3	14.6–22.7	32.5	27.8–37.7	25.1	20.7–30.1	49.3	43.9–54.8	10.6	7.6–14.6	17.6	14.0–22.0
45–59	42.6	36.6–48.7	35.5	30.0–41.4	19.7	15.4–24.9	45.8	39.8–51.9	12.5	8.8–17.5	12.0	8.8–16.1
60+	65.0	59.8–69.9	35.1	30.3–40.2	24.4	20.2–29.2	42.3	37.1–47.6	20.9	17.0–25.4	7.9	5.7–10.8
Gender												
Male	40.7	36.2–45.3	28.7	24.9–32.9	19.4	16.0–23.2	39.8	35.3–44.4	11.4	8.9–14.5	10.6	8.2–13.7
Female	33.7	30.0–37.8	36.9	32.9–41.0	27.6	23.9–31.5	49.3	45.1–53.6	14.7	12.0–17.9	18.4	15.3–21.9
Race/Ethnicity												
White, NH	37.1	33.6–40.7	34.1	30.7–37.7	26.3	23.1–29.8	46.7	43.0–50.4	14.6	12.2–17.5	15.9	13.3–19.0
Black, NH	47.5	38.6–56.5	31.5	24.2–39.8	22.3	16.2–29.8	40.6	32.3–49.6	11.9	7.2–19.1	9.7	6.2–14.8
Hispanic	26.5	19.3–35.3	32.4	25.0–40.9	17.7	11.8–25.6	41.6	33.2–50.6	10.2	6.2–16.3	11.9	8.0–17.3
Other, NH	43.3	32.6–54.7	27.3	18.6–38.2	17.5	10.5–27.6	42	31.4–53.4	9.2	5.1–16.2	17.3	10.1–28.1
Education												
No HS diploma	43.8	31.3–57.2	23.1	14.5–34.7	34.0	22.8–47.2	45.8	33.2–58.9	17	10.0–27.5	22.6	13.9–34.7
HS graduate	38.8	32.5–45.5	33.8	27.7–40.5	23.1	17.9–29.3	40.7	34.1–47.6	13.4	9.6–18.6	12.5	8.7–17.6
Some college	39.9	35.5–44.5	36.9	32.6–41.3	25.8	22.0–30.1	49.5	44.9–54.0	13.8	11.1–17.0	16.2	13.2–19.6
Bachelors or above	31.5	27.2–36.1	31.9	27.6–36.5	19.3	15.9–23.1	43.9	39.1–48.8	11.2	8.4–14.8	12.9	9.8–16.7
Household income												
< $30,000	45.8	39.6–52.1	33.4	27.9–39.4	28.3	23.0–34.1	45.5	39.3–51.9	15.8	12.2–20.3	18.9	14.9–23.6
$30,000 to <$50,000	35.0	28.4–42.3	35.2	28.7–42.3	26.6	20.7–33.5	43.1	36.1–50.4	14.3	9.8–20.4	17.5	12.6–23.7
$50,000 to < $75,000	37.6	31.0–44.8	36.3	29.9–43.2	22.7	17.4–29.0	46.7	39.6–53.8	15.1	10.6–21.1	18.7	13.1–26.1
$75,000 to < $100,000	28.9	21.9–37.0	30.9	23.9–38.8	22.3	15.7–30.8	42.6	34.4–51.2	8	5.1–12.2	11.4	7.2–17.6
≥ $100,000	34.3	28.5–40.5	29.3	24.0–35.2	17.6	13.4–22.8	44.9	38.8–51.2	11	7.4–16.0	6.4	4.3–9.4
Population density												
Rural	35.5	26.6–45.5	35.9	26.8–46.2	30.2	21.6–40.4	50	39.9–60.1	12.2	7.7–18.9	17.3	10.7–26.9
Suburban	38.7	32.4–45.3	38.0	31.6–44.8	26.3	20.8–32.7	44.6	38.1–51.4	13.9	10.2–18.6	15.7	11.6–21.0
Urban	36.9	33.3–40.6	31.2	27.9–34.6	21.9	19.0–25.2	43.9	40.2–47.7	13	10.7–15.7	13.9	11.6–16.7

CI, confidence interval.

### COVID-19 preventive behaviors by chronic conditions

As displayed in Table 3, significant differences in the prevalence of COVID-19 related preventive behaviors were observed across categories of chronic conditions. Compared with individuals without cardiometabolic disease, among adults with cardiometabolic disease, significant differences were observed in their reported prevalence of canceling or postponing a variety of activities, including cancelation of a doctor appointment (*p*=0.002, 28.8% vs. 38.4%, respectively), cancelation of work activities (*p*<0.001, 38.1% vs. 22.9%), and cancelation of school activities (*p*<0.001, 25.5% vs. 14.0%). Adults with cardiometabolic disease were significantly less likely to work (24.4% vs. 36.8%, *p*<0.001) or study (9.2% vs. 18.1%, *p*<0.001) from home. Adults with cardiometabolic disease were also significantly more likely to report wearing a face mask (81.9% vs. 75.0%, *p*=0.013), visiting a doctor or hospital (11% vs. 6.0%, *p*=0.007), and praying (63.6% vs. 51.6% prevalence, *p*<0.001).

**Table 3.** Behaviors Due to COVID-19 Pandemic by Chronic Conditions

	Cardiometabolic disease			Overweight			Respiratory			Allergies			Immune			Mental health		
	%	95% CI	p	%	95% CI	p	%	95% CI	p	%	95% CI	p	%	95% CI	p	%	95% CI	p
Washed or sanitized hands	91.2	86.6–94.3	0.687	95.9	93.5–97.5	0.001	93.4	87.9–96.5	0.402	94.5	91.4–96.5	0.018	95	90.4–97.5	0.117	94.0	89.3–96.7	0.27
Keep six feet distance from those outside my household	85.1	80.4–88.8	0.811	87.7	83.9–90.8	0.167	85.9	79.9–90.3	0.848	89.2	85.6–92.1	0.007	87.9	80.9–92.5	0.423	84.8	78.3–89.6	0.789
Wore a face mask	81.9	77.6–85.4	0.013	76.6	71.7–80.9	0.646	78.3	72.3–83.4	0.743	81.1	77.3–84.3	0.016	86.7	80.4–91.2	0.004	75.3	67.7–81.6	0.49
Avoided public or crowded places	81.8	77.6–85.3	0.436	84.2	80.6–87.3	0.022	81.2	76.3–85.4	0.724	86.3	83.1–88.9	<0.001	82.8	75.6–88.2	0.461	82.9	76.4–87.9	0.407
Avoided some or all restaurants	72.7	67.9–77.0	0.561	73.1	68.5–77.3	0.435	71.7	65.9–76.8	0.972	73.3	69.1–77.1	0.286	79.2	70.9–85.5	0.048	75.2	68.1–81.2	0.266
Canceled or postponed pleasure, social, or recreational activities	66.0	60.8–70.8	0.107	73.7	69.1–77.8	0.025	70.8	64.4–76.4	0.559	74.6	70.4–78.5	0.001	68.2	59.7–75.7	0.812	70.1	62.5–76.7	0.794
Prayed	63.6	58.6–68.3	<0.001	61.7	56.5–66.6	0.011	58.8	52.4–64.9	0.332	59.3	54.6–63.7	0.068	68.9	60.9–75.9	0.001	54.0	46.0–61.8	0.579
Avoided contact with high-risk people	62.7	57.6–67.5	0.784	64.4	59.4–69.1	0.296	67.2	60.9–72.9	0.072	67.4	63.0–71.6	0.002	69.7	61.7–76.6	0.047	74.1	67.2–80.0	<0.001
Wiped packages entering my home	46.6	41.6–51.7	0.534	46.1	41.1–51.3	0.718	47.2	40.9–53.6	0.506	48.8	44.2–53.4	0.052	50	41.8–58.2	0.242	45.3	37.5–53.5	0.999
Canceled a doctor appointment	38.4	33.6–43.5	0.002	33.2	28.7–38.1	0.690	34.0	28.1–40.3	0.559	36.0	31.7–40.6	0.028	36.9	29.6–44.9	0.212	35.1	27.8–43.2	0.444
Canceled or postponed dentist or other appointment	33.0	28.6–37.8	0.121	37.6	32.8–42.6	0.465	35.5	29.8–41.7	0.855	40.3	35.9–44.8	0.012	38.8	31.2–46.9	0.456	38.8	31.2–47.0	0.452
Stockpiled food or water	29.3	25.0–34.1	0.101	36.2	31.4–41.2	0.070	31.7	26.0–38.0	0.774	34.8	30.5–39.3	0.157	31.7	24.6–39.9	0.843	32.1	25.2–39.8	0.915
Worked from home	24.4	20.4–29.0	<0.001	34.3	29.7–39.3	0.295	35.5	29.6–41.9	0.212	35.9	31.6–40.4	0.021	27.7	20.5–36.1	0.242	38.4	30.8–46.6	0.086
Canceled or postponed work activities	22.9	18.9–27.4	<0.001	30.2	25.6–35.3	0.290	37.3	31.2–43.8	0.074	35.3	31.0–39.9	0.087	24.9	18.3–32.9	0.045	34.7	27.2–43.2	0.536
Canceled or postponed school activities	14.0	10.6–18.2	<0.001	18.6	15.0–22.8	0.150	25.9	20.3–32.5	0.056	23.2	19.5–27.3	0.181	13.2	8.8–19.5	0.01	23.5	17.5–30.7	0.458
Visited a doctor or hospital	11.1	8.3–14.5	0.007	7.3	5.2, 10.1	0.630	9.7	6.8–13.8	0.212	8.1	6.0–10.9	0.821	9.1	5.5–14.6	0.561	11.9	7.9–17.3	0.038
Stayed home because I felt unwell	10.6	8.0, 13.8	0.967	13.5	10.3–17.4	0.037	19.4	14.6–25.3	<0.001	12.9	10.3–16.2	0.036	11.8	8.2–16.8	0.564	18.9	13.4–26.2	<0.001
Canceled outside housekeepers or caregivers	9.2	6.7–12.5	0.112	9.5	7.0–12.8	0.188	11.5	7.5–17.4	0.933	11.2	8.3–15.0	0.931	14.1	8.8–21.8	0.336	10.4	6.3–16.7	0.712
Studies from home	9.2	6.9–12.1	<0.001	13.8	10.6–17.8	0.534	15.7	11.8–20.5	0.661	16.0	12.7–19.8	0.378	14.1	9.5–20.5	0.799	18.0	12.6–25.1	0.243

Adults who were overweight/obese were significantly more likely to report avoiding public or crowded places, compared with adults who were normal weight (84.2% vs. 78.7%, *p*=0.022) and were more likely to report washing or sanitizing their hands, compared with adults who were normal weight (95.9% vs. 89.7%, *p*<0·001). Adults who were overweight or obese also had a significantly higher prevalence of staying at home because they felt unwell, compared with adults who were normal weight (13.5% vs. 9.2% prevalence, *p*=0.037).

Adults with respiratory conditions had a significantly higher prevalence of staying at home because they felt unwell, compared with adults without respiratory conditions (19.4% vs. 7.9% *p*<0.001). Compared with individuals without allergies, significant differences were observed among adults with allergies in their reported prevalence of canceling or postponing a variety of activities, including cancelation of a doctor appointment (36.0% vs. 29.5%, *p*=0.028) cancelation of recreational or social activities (74.6% vs. 64.7%, *p*=0.001), and cancelation of dental appointments (40.3% vs. 32.6%, *p*=0.012). Adults with allergies were also significantly more likely to report wearing a face mask (81.1% vs. 74.6%, *p*=0.016), avoiding public or crowded places (86.3% vs. 75.8%, *p*<0.001), maintaining six feet of social distance (89.2% vs. 82.5%, *p*=0.007), avoiding contact with high-risk people (67.4% vs. 57.8%, *p*=0.002), and washing or sanitizing their hands (94.5% vs. 89.5%, *p*=0.018).

Adults with immune conditions had a significantly higher prevalence of wearing a face mask compared with those without immunocompromised conditions (86.7% vs. 76.1% *p*=0.004). Adults with immune conditions had a significantly lower prevalence of canceling work (24.9% vs. 33.6%, *p*=0.045) or school activities (13.2% vs. 22.4%, *p*=0.01, respectively), compared with adults without underlying immune conditions. Adults with immune conditions had a significantly higher prevalence of avoiding restaurants (79.2% vs. 70.4%, *p*=0.048) and avoiding contact with high-risk people (69.7% vs. 61.0%, *p*=0.047, respectively), compared with adults without underlying immune conditions.

Adults with mental health conditions had a significantly higher prevalence of visiting a doctor or hospital (11.9% vs. 7.2%, *p*=0.38), avoiding contact with high-risk people (74.1% vs. 60.1%, *p*<0.001), and staying home because they felt unwell (18.9% vs. 9.2%, *p*<0.001).

### Multivariable associations of COVID-19-related behaviors by chronic disease conditions

Table 4 displays the results from the multivariable logistic regression models for each chronic health condition and COVID-19-related behavior adjusting for age, gender, race, and educational status. In fully adjusted models, adults with cardiometabolic disease were 1.6 times as likely to report staying home because they felt unwell, compared with individuals without cardiometabolic disease (aOR: 1.63, 95% CI 1.05–2.53). Adults who were overweight or obese were 2.3 times as likely to report washing or sanitizing their hands (aOR: 2.28, 95% CI 1.24–4.19), and were 1.6 times as likely to report staying home because they felt unwell (aOR: 1.63, 95% CI 1.06–2.50), when compared with those who were normal weight. Individuals with underlying respiratory conditions were 1.6 times as likely to work from home, compared with individuals without an underlying respiratory condition (aOR: 1.67, 95% CI 1.17–2.38) and were nearly 3 times as likely to stay home because they felt unwell (aOR: 2.87, 95% CI 1.86–4.42), when compared with individuals without an underlying respiratory condition.

**Table 4.** Multivariable Associations of COVID-19-Related Behaviors by Chronic Disease Conditions^a^

	Cardiometabolic disease		Overweight		Respiratory		Allergies		Immune		Mental health	
	OR	95% CI	OR	95% CI	OR	95% CI	OR	95% CI	OR	95% CI	OR	95% CI
Worn a face mask	1.19	0.85–1.66	0.89	0.64–1.24	1.21	0.84–1.74	1.53	1.12–2.10	1.98	1.19–3.28	1.08	0.69–1.69
Avoided some or all restaurants	0.95	0.70–1.30	1.08	0.81–1.45	1.09	0.78–1.51	1.18	0.88–1.57	1.59	0.98–2.57	1.46	0.96–2.20
Worked from home	0.77	0.52–1.13	1.22	0.92–1.63	1.67	1.17–2.38	1.45	1.08–1.95	1.10	0.66–1.81	1.56	1.04–2.34
Canceled or postponed pleasure, social, or recreational activities	0.73	0.53–1.00	1.27	0.93–1.72	1.10	0.79–1.52	1.53	1.14–2.06	0.86	0.57–1.31	1.06	0.70–1.60
Avoided public or crowded places	0.91	0.65–1.28	1.37	0.99–1.89	1.10	0.76–1.59	2.06	1.50–2.83	1.06	0.66–1.70	1.37	0.85–2.21
Avoided contact with high-risk people	1.02	0.75–1.38	1.08	0.83–1.42	1.31	0.95–1.79	1.46	1.12–1.92	1.40	0.95–2.07	1.94	1.33–2.85
Washed or sanitized hands	0.68	0.39–1.17	2.28	1.24–4.19	1.37	0.75–2.50	1.89	1.07–3.34	1.28	0.57–2.85	1.75	0.77–3.97
Keep six feet distance from those outside my household	0.56	0.36–0.86	1.14	0.76–1.71	1.09	0.69–1.75	1.75	1.16–2.64	0.94	0.53–1.68	1.23	0.70–2.16
Stayed home because I felt unwell	1.63	1.05–2.53	1.63	1.06–2.50	2.87	1.86–4.42	1.55	1.00–2.39	1.41	0.87–2.29	1.90	1.14–3.17
Wiped packages entering my home	0.97	0.72–1.31	1.03	0.79–1.35	1.14	0.85–1.55	1.30	1.00–1.69	1.18	0.81–1.73	1.08	0.74–1.56

^a^ Models adjusted for age, gender, educational status, race (White, non-Hispanic/non-White)

OR, odds ratio.

Adults with allergies were 1.5 as likely to report wearing a face mask compared with individuals without allergies (aOR: 1.53, 95% CI 1.12–2.10). Adults with allergies were 1.4 times as likely to report working from home (aOR: 1.45, 95% CI 1.08–1.95), 1.5 times as likely to report canceling or postponing social activities (aOR: 1.53, 95% CI 1.14–2.06), and more than twice as likely to avoid public or crowded places (aOR: 2.06, 95% CI 1.50–2.83), compared with individuals without allergies. Adults with allergies were nearly 1.5 times as likely to avoid contact with high-risk people (aOR: 1.46, 95% CI 1.12–1.92), nearly twice as likely to wash or sanitize their hands (aOR: 1.89, 95% CI 1.07–3.34), and nearly twice as likely to report maintaining six feet of social distance (aOR: 1.75, 95% CI 1.16–2.64), compared with individuals without allergies.

Adults with immune conditions were twice as likely to report wearing a face mask when compared with individuals without immune conditions (aOR: 1.98, 95% CI 1.19–3.28). Adults with a mental health condition were likely to report working from home compared with those without a mental health condition (aOR: 1.56, 95% CI: 1.04–2.34). Also, adults with mental health conditions were nearly twice as likely to report avoiding contact with high-risk people (aOR: 1.94, 95% CI 1.33–2.85) and nearly twice as likely to report staying home because they felt unwell (aOR: 1.90, 95% CI 1.14–3.17), compared with adults without a mental health condition.

## Discussion

Results from this study provide U.S. national prevalence estimates and differences in adherence to COVID-19 preventive behaviors among those with and without the presence of underlying chronic health conditions. The prevalence of key preventive measures, including washing or sanitizing their hands, use of a face mask, and maintaining appropriate social distancing in public was high in the overall sample. Yet, engagement in COVID-19-related preventive behaviors varied significantly across chronic disease conditions. The use of face masks in public were only more common among individuals with allergies or underlying immune conditions.

Our findings are of importance in the context of recent reports underscoring potential compounded vulnerability among those with cardiometabolic-related disease.^16^ Emerging international and domestic evidence has identified hypertension, cardiovascular disease, obesity, and diabetes as key risk factors, aside from age, for COVID-19 incidence and mortality.^9,16–19^ For example, a retrospective case series of 1591 laboratory-confirmed COVID-19 in Italy identified hypertension, cardiovascular disease, and hypercholesterolemia as the most common comorbidities among cases. Similar results were reported in a case series analysis of 5700 hospitalized adults in NY with confirmed COVID-19; however, after hypertension, the most common comorbidities were obesity and diabetes.^20^ The current analysis of a U.S. representative population identified allergies, cardiometabolic diseases, and overweight or obesity as the most reported comorbidities. Interestingly, adults in the present sample with underlying cardiometabolic disease, respiratory disease, or obesity were significantly more likely to report staying home because they felt unwell, compared with individuals without these conditions.

Interestingly, individuals with allergies were more likely to engage in nearly all COVID-19 preventive behaviors, compared with individuals without allergies. This could be due to seasonality issues of the COVID-19 pandemic overlapping with increased presence of environmental allergens. Symptoms associated with allergies, such as a runny nose, watery eyes, and sneezing may make individuals with allergies more likely to engage in COVID-19 preventive behaviors to avoid touching their faces in public. The World Health Organization has released multiple reports advising against touching face in effort to reduce transmission of the virus, as well as the reinforcement of preventive behaviors given overlapping symptoms between allergies and COVID-19.^3^

Our results should be interpreted within the context of limitations. First, the survey field period was between April 20 and 26, 2020. There may also be potential for social desirability bias in terms of report of COVID-19 preventive behaviors. Data available in this analysis did not include occupational categories, household size, or whether household members were following preventive behaviors. Some behaviors related to report of working from home may reflect opportunity and privilege, rather than the willingness of the individual to engage in the behavior.^21,22^ Recent reports have suggested a disproportionate impact of COVID-19 pandemic by social determinants of health, including characteristics such as immigration status and history, household size, and composition; therefore, future studies should consider inclusion of these variables.^23,24^

## Conclusion

Despite limitations, our study has notable strengths. First, sampling methods used to obtain a nationally representative sample of the U.S. adult population provides the opportunity for increased generalizability. Findings may be helpful to clinicians and public health officials to guide clinical and public health messaging as many states within the U.S. join other countries in preparation for a potential second outbreak of cases in the Fall. Additionally, this study includes a broad range of COVID-19-related behaviors, including those not typically endorsed by WHO and related health agencies (i.e., prayer). Importantly, results are drawn from a population that is diverse across social and demographic characteristics as well as health status. Considering that the pandemic has exacerbated inequities by social and structural determinants of health, public health strategies that are culturally and structurally competent and that which consider the nuances of local communities will have a vital role in reducing COVID-19 disparities.^7,8,21,22,24,25^ Study implications suggest a need for more targeted messaging and resources available for individuals with certain underlying chronic conditions, particularly adults with cardiometabolic disease, obesity, or respiratory diseases.^10,16,19,26^

## Abbreviations Used

aOR: adjusted odds ratio
CI: confidence interval
COPD: chronic obstructive pulmonary disease
HS: high school
NH: non-Hispanic
OR: odds ratio
SARS-CoV-2: severe acute respiratory syndrome coronavirus 2
